# The effect of nocturnal wear of complete dentures on sleep and oral health related quality of life: study protocol for a randomized controlled trial

**DOI:** 10.1186/1745-6215-15-358

**Published:** 2014-09-13

**Authors:** Elham Emami, Phan The Huy Nguyen, Fernanda R Almeida, Jocelyne S Feine, Igor Karp, Gilles Lavigne, Nelly Huynh

**Affiliations:** Faculty of Dentistry, Université de Montréal, 2900 Edouard-Montpetit, Montreal, QC H3T 1J4 Canada; Faculty of Dentistry, McGill University, 3550 University Street, Montreal, QC H3A 2A7 Canada; Faculty of Dentistry, University of British Colombia, #103 - 2786W 16th Ave, Vancouver, BC V6K 4M1 Canada; Schulich School of Medicine and Dentistry, University of Western Ontario, 1151 Richmond Street, London, ON N6A 5C1 Canada

**Keywords:** Complete denture, edentulism, obstructive sleeping apnea, oral health related quality of life, sleep disorders, sleep quality

## Abstract

**Background:**

Edentulism and sleep disturbance are chronic conditions that are common in older people and have serious adverse consequences for their functioning and quality of life. Edentulism can disturb sleep through the alteration of the craniofacial structure and surrounding soft tissue. However, the effect of prosthetic rehabilitation of edentulism on sleep quality is still not well understood. The objectives of this study are to test whether nocturnal denture wear affects sleep quality, daytime sleepiness, and the oral health related quality of life of edentate older people with moderate to severe sleep apnea, and to identify modifiers of effect of nocturnal denture wear.

**Methods/design:**

We will carry out a single-blind randomized cross-over trial. Seventy edentate older people with moderate to severe obstructive sleep apnea will be enrolled. The study participants will be assigned to wear and not wear their dentures on alternate periods of 30 days. The outcome measures will be sleep quality (assessed by portable polysomnography), daytime sleepiness (assessed by the Epworth Sleepiness Scale), and oral health related quality of life (assessed by validated questionnaire). A number of characteristics (sociodemographic, oropharyngeal morphology, oral and prosthesis characteristics, and perceived general health quality of life) will be assessed by means of clinical examination, 3D imaging of the craniofacial structure, and validated questionnaires at baseline. Linear mixed effects regression models for repeated measures will be fitted to test the study hypotheses. The main analyses will be based on the intention-to-treat principle. To assess the robustness of the findings to potential incomplete adherence, sensitivity analyses will be conducted while applying the per-protocol principle.

**Discussion:**

This practice-relevant evidence could represent a preventive approach to improve sleep characteristics of the older population and improve their well-being and quality of life.

**Trial registration:**

ClinicalTrials.gov NCT01868295.

## Background

The worldwide population is rapidly aging, with the majority of older people living longer than previous generations [1]. Aging substantially increases the risk of tooth loss and sleep disturbance. In Canada, about one in four people aged 65 and older is completely toothless [2], and nearly half of all older people complain of disturbed sleep [3, 4]. These two chronic conditions have serious adverse consequences for well-being, functioning, and quality of life, and place a significant burden on the health care system [5–8]. The costs associated with sleep disturbance and tooth loss are also substantial [7, 9–12].

The relationship between edentulism and sleep disturbance and sleep-disordered breathing has been well documented [13, 14]. In fact, anatomical changes associated with edentulism and decrease in the vertical dimension of occlusion can negatively influence sleep and lead to obstructive sleep apnea [13–18]. Loss of the vertical dimension of occlusion leads to a forward and upward mandibular position associated with a backward shift in the supine position [16, 19]. This rotational movement of the mandible favors a shift of the tongue and soft palate against the posterior pharyngeal wall. The reduction in the retropharyngeal space associated with impaired function of the upper airway dilatation muscles results in upper airway resistance, and diminished response to negative pressure stimulation [20–22]. Moreover, age-specific compromised pharyngeal anatomy, upper airway mucosal sensory dysfunction, and a decline in pharyngeal sensory discrimination and reflexes have been proposed as being responsible for the vulnerability of edentate elders to airway collapse [23–25]. Furthermore, the literature suggests that there is a possible association between sleep disturbance and wearing a complete denture during sleep, which may explain the mechanism underlying the relationship between obstructive sleep apnea and edentate individuals [26, 27]. One hypothesized effect was that the use of a denture during sleep may lead to open bite and mouth breathing with a decrease in the tone of the pharyngeal muscles, thus leading to the development or worsening of obstructive sleep apnea [28]; an alternative hypothesis holds that sleep quality and pharyngeal patency are maintained by nocturnal denture use. Moreover, this denture effect appears to be different between healthy edentate older people and those with sleep disturbances [29]. However, there is a general belief amongst oral health care professionals that edentate individuals should remove their dentures at night. In fact, numerous studies have demonstrated that long-term nocturnal wearing of dentures can reduce the protective effect of saliva and obstruct good oxygenation of the oral mucosa, which makes it less resistant to mechanical and microbiological aggression, thus increasing the risk of chronic inflammatory changes within the mucosa [30–32]. This leads to an increased risk of traumatic ulcers, denture stomatitis, and alveolar bone resorption in the edentate population [33–37].

However, tooth loss and not wearing dentures at night have not been recognized as a risk factor for sleep disturbance and obstructive sleep apnea. Furthermore, the limited quality of studies on this topic (graded as level ≤3 on the Oxford level of grade of evidence) [38] do not permit clinicians to engage in evidence-based clinical decision-making. This lack of knowledge poses legal and ethical problems for clinicians and other dental professionals who are involved in the care of the growing edentate population.

To enable development of clinical practice guidelines, solid evidence is required. Specifically, a rigorous randomized trial is needed to help determine the effects of denture wearing on patient-relevant outcomes [28, 39–41].

To address this need, we have designed a single-blind randomized cross-over trial. The first objective of the study is to test whether nocturnal denture wear has an effect on sleep quality and daytime sleepiness of edentate older people with moderate to severe sleep apnea. The second objective is to test whether nocturnal denture wear has an effect on the oral health related quality of life of edentate older people with moderate to severe sleep apnea. The third objective is to identify modifiers of the putative effects of nocturnal denture wear.

## Methods/design

The study protocol was approved by the Research Ethics Board of Health of the Université de Montréal (Project 13-076-CERES-D). The trial is registered in the US Clinical Trials Registry http://NCT01868295.

### Recruitment process

Participants will be recruited from the area of metropolitan Montreal. Participants will be recruited through the research, prosthodontics, and sleep apnea clinics of the Université de Montréal, associated hospitals, geriatric institutes, and private sleep clinics.

The research coordinator will contact the patients in person at the clinics with a study information brochure to invite them to participate in the trial. Potential study participants will be asked to contact a research coordinator via a dedicated phone number with voicemail. The research coordinator will describe the study in general terms and assess major inclusion criteria, and the study candidates will be invited to an in-person information and screening session. During this session, the research coordinator will inform these potential participants of the general health risks associated with obstructive sleep apnea and will explain all aspects of the study, using a PowerPoint presentation and a study brochure. The interested participants will be screened for eligibility for inclusion in the study. Eligible participants will be asked to read and sign a consent form. Informed consent will be obtained from each eligible participant before proceeding with the trial.

### Inclusion and exclusion criteria

#### Inclusion criteria

To be considered for inclusion in the study, the subject must: (1) be aged 65 years or older; (2) have worn a complete set of removable dentures in the past year but not have worn the dentures during sleep in that period; (3) have an Apnea-Hypopnea Index (AHI) score of at least 15 at screening; (4) have an adequate understanding of written and spoken English or French; (5) be able to understand and respond to the questionnaires used in the study; (6) agree to follow the research study instructions; (7) agree to adhere to the allocated sequence of interventions; (8) consume no alcohol and (9) not work late at night on the day before polysomnography.

#### Exclusion criteria

The participants are excluded if they: (1) have an AHI score less than 15; (2) have any severe cardiologic, neurologic, psychological, or psychiatric condition, respiratory disease, acute airway infection, or any other health condition that jeopardizes sleep; (3) score 24 or less on the Mini-Mental State Evaluation [42]; (4) regularly consume more than two (for women) or three (for men) alcoholic beverages per day; (5) are taking medication or any illicit drug that will affect sleep architecture or respiratory muscle activity (that is, hypnotics, psychostimulants, anticonvulsant, or antipsychotics); (6) are receiving regular continuous positive airway pressure therapy or nocturnal supplemental oxygen; (7) have sleepiness deemed to be unsafe and requiring urgent treatment; or (8) feel that the trial would negatively influence their private life.

### Intervention, randomization, allocation concealment, and sequence generation

A single-blind randomized cross-over clinical trial will be conducted. The trial will have two sequences and two periods (balanced design).

**Figure 1:**
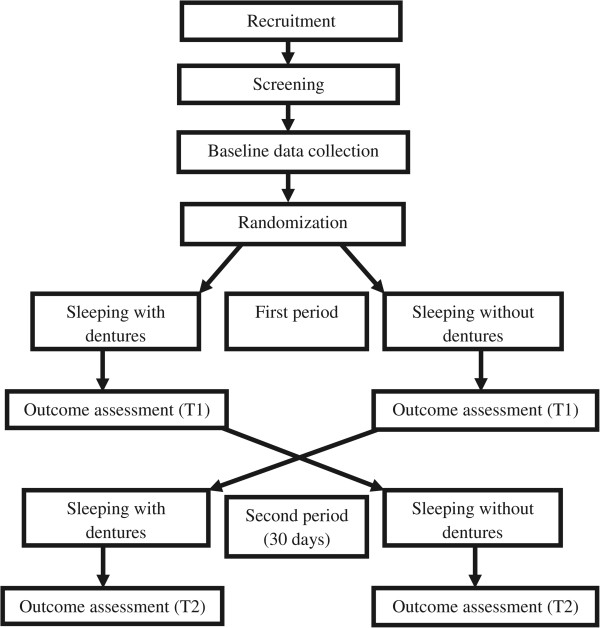
**Trial design.**

Eligible study participants will be randomly assigned to wear or not wear their complete dentures at night, alternately, for two periods of 30 days (Figure 1). Minimal or no carry-over effect is expected based on the action of the postulated mechanism of intervention [28] (additional analyses of raw data from research team member FRA: Grizzle’s model [43] sequence effect *P* = 0.8, period effect *P* = 0.9, intervention effect *P* = 0.002). Therefore, no wash-out period was deemed necessary.

To achieve a balanced allocation of participants in each sequence, a permuted block randomization with varying block sizes using SAS® PROC PLAN will be used [44–51]. Randomization and administration of opaque sequential envelopes will be carried out off-site. The sleep technologist will deliver these envelopes to study participants at their homes. Each participant will receive a sequentially numbered, sealed, opaque, tamper-proof envelope indicating the sequence of intervention, and intervention will start on the same day (one week after baseline assessment). All the investigators, the research coordinator, and the scoring service providers will be blinded to the intervention assignment. Data will be gathered, measured, recorded, and entered in a blinded fashion. Blinding will be lifted once data analysis is complete. Although it is not possible for the participants to be ‘blind’ to the intervention, they will be asked not to discuss their interventions with any research staff. Any question from participants during the intervention period will be answered by two independent prosthodontists and, if necessary, a sleep clinician, as these individuals will not be involved in data collection or data analyses.

### Data collection

Persons who consent to participate in the study will be invited to visit the clinical research laboratories of the Faculty of Dental Medicine at the Université de Montréal at their convenience. During this visit, one trained and calibrated research trainee will conduct baseline data collection, including a clinical examination, administration of study questionnaires, and cone-beam computed tomography (CBCT) (NewTom 5G CBCT, QR S.r.l.-Verona, Italy), as described. Then, in the same week, they will undergo one baseline portable overnight recording (level II polysomnography) [52]. Follow-up data will be collected at the end of each period by the sleep technologist at home by means of one portable overnight recording and the outcome questionnaires. The sleep technologist will install the device at the participants’ homes on the evening of recording, verifying electrode impedance and signal quality. This approach will allow us to maximize the participation of older people and will increase their adherence to the study procedures [53].

#### Primary outcome measures

The selection of the outcomes was based on a literature review and consultation with experts in sleep and oral health disease [54, 55].

The primary outcome is a change in sleep quality, as measured by the AHI index. This index is a marker of sleep quality that represents the number of apneic and hypopneic incidents per hour of sleep [56]. The AHI index will be measured using diagnostic portable polysomnography units for overnight home use [57]. Standard polysomnography measurements will include total sleep duration, sleep efficiency, sleep onset latency, electro-encephalogram arousal index, spontaneous arousals, and sleep-stage distribution. Upon return of the device, polysomnographic data will be downloaded and scored using REMLogic software (Embla Inc., Canada) to assess AHI, snoring index, respiratory disturbance index, flow limitation, respiratory efforts related arousals, and average and minimal oxygen saturation. These respiratory events will be scored by Sleep Strategies Inc. (Canada), according to American Academy of Sleep Medicine guidelines [58].

#### Secondary outcome measures

Secondary outcomes will be changes in daytime sleepiness and oral health related quality of life.

The Epworth Sleepiness Scale (ESS) will be used to assess perceived daytime sleepiness. The ESS is an eight-item, four-point scale (0 to 3) with strong internal consistency (Cronbach’s α = 0.81) and half-split reliability (*r* = 0.82) [59]. Participants will be asked to rate their chance of dozing in eight different sedentary situations. Scores of 10 or more suggest excessive daytime sleepiness.

Oral health related quality of life will be measured by means of the Oral Health Impact Profile (OHIP-20) [60]. This instrument is a disease-specific measure of people’s perceptions of the impact of denture wear on physical, psychological, and social aspects of their quality of life. This validated and highly reliable (α = 0.88) oral health disease-specific instrument has been widely used in geriatric dental research [61–63] and has been tested and validated in English- and French-speaking Canadians for cross-cultural validity. The range of the scale is 20 to 120 points, with lower scores indicating a better oral health related quality of life [60, 64].

The primary and secondary outcome measures will be determined at baseline and at the end of each 30-day period.

### Covariates

We will also collect data on sociodemographic, medical, and anthropometric (weight, height) characteristics, oropharyngeal morphology (measured by a three-dimensional imaging system, CBCT), as well as edentulism-associated characteristics (vertical dimension of occlusion according to prosthodontic standard criteria, history of tooth loss, history of denture use, and nocturnal denture wear) [65] and general health. Perceived general health will be assessed using the Short Form-36 (SF-36) [66]. This is a generic self-administered questionnaire consisting of eight multi-item subscales: physical functioning, social functioning, role limitations due to physical health problems, and role limitations due to emotional problems, mental health, vitality, pain, and general health perceptions. The SF-36 has excellent internal consistency and discriminates between individuals with and without chronic disease [66–68]. The computerized scoring system will be used according to the user’s manual, in which higher scores represent a better condition [69].

### Sample size justification

Assuming that (i) the minimal clinically important difference in the AHI score between the two interventions is 5 events per hour (based on the opinion of expert clinicians, Delphi method) [70], (ii) the standard deviation of the distribution of the difference in AHI score between interventions is 10.6 events per hour (based on estimates from our pilot data) [28], and (iii) the drop-out rate is 10% (based on our pilot data), a sample size of 70 study participants will ensure a 0.90 power to reject the null hypothesis if it is indeed false, at a two-sided Bonferroni-adjusted α level of 0.0167 (to account for the three study outcomes).

The sample size of 70 would ensure a power of more than 0.90 to detect a difference of 2.5 units in the mean ESS score (assuming that the standard deviation is 3.5 units) [71], and a difference of 17 units (minimal clinically important difference) [72] in the mean OHIP score (assuming that the standard deviation is 23.8 units) [73], at a two-sided Bonferroni-adjusted α level of 0.0167 [74, 75].

### Statistical analysis

Descriptive analyses for all variables will be performed. In case of apparent material deviations from normality, the distributions of the dependent variables will be normalized by appropriate transformations.

Mixed linear models will be fitted using SAS Proc Mixed (SAS version 9.3, SAS Institute Inc., USA) to test the associations between the intervention and each of the study outcomes [76]. The model will include variables for intervention, randomization sequence, period, and baseline value of the study outcome as fixed effects and subject as a random effect. Hypotheses concerning model parameters will be tested by Wald tests with an approximate *F* null distribution.

To test for potential modification of the impact of the intervention by determining the presence of sleep disturbance at baseline, and the degree of sleep disturbance at baseline, we will introduce a series of interaction terms between the intervention status and each of these characteristics (one at a time) into the above-described linear models.

All of the analyses will be carried out in accordance with the intention-to-treat principle, and various strategies will be contemplated, to account for post-randomization missing data in the case of non-balanced missing data or attrition of more than 10% of participants. However, in the sensitivity analyses, we will also carry out per-protocol analyses to assess the robustness of our findings to potential incomplete adherence: in particular, patients with major protocol violations or poor adhere will be excluded.

## Discussion

This research could inform planning for a preventive approach to improve sleep characteristics of the older population and, thereby, improve well-being and quality of life. The evidence produced by this trial will assist in producing truly evidence-based practice guidelines used by primary care providers, dentists, and sleep medicine specialists, who provide care for the millions of edentate older people around the world [56].

### Trial status

The project is currently in the recruitment phase.

## Abbreviations

AHI: Apnea-Hypopnea Index
CBCT: cone-beam computed tomography
ESS: Epworth Sleepiness Scale questionnaire
OHIP-20: Oral Health Impact Profile questionnaire
SF-36: Short Form-36 Health Survey questionnaire.
